# Differential plasticity of excitatory and inhibitory reticulospinal fibers after spinal cord injury: Implication for recovery

**DOI:** 10.4103/NRR.NRR-D-24-01060

**Published:** 2025-02-24

**Authors:** Rozaria Jeleva, Carmen Denecke Muhr, Alina P. Liebisch, Florence M. Bareyre

**Affiliations:** 1 Institute of Clinical Neuroimmunology, University Hospital, LMU Munich, Munich, Germany; 2 Biomedical Center Munich (BMC), Medical Faculty, LMU Munich, Planegg-Martinsried, Germany; 3 Graduate School of Systemic Neurosciences, Ludwig-Maximilians-Universitaet Munich, Planegg-Martinsried, Germany; 4 Munich Cluster of Systems Neurology (SyNergy), Munich, Germany

**Keywords:** GABAergic (vGat) fibers, gait features, glutamatergic (vGlut2) fibers, plasticity, recovery of function, reticulospinal tract, spinal cord injury

## Abstract

The remodeling of axonal connections following injury is an important feature driving functional recovery. The reticulospinal tract is an interesting descending motor tract that contains both excitatory and inhibitory fibers. While the reticulospinal tract has been shown to be particularly prone to axonal growth and plasticity following injuries of the spinal cord, the differential capacities of excitatory and inhibitory fibers for plasticity remain unclear. As adaptive axonal plasticity involves a sophisticated interplay between excitatory and inhibitory input, we investigated in this study the plastic potential of glutamatergic (vGlut2) and GABAergic (vGat) fibers originating from the gigantocellular nucleus and the lateral paragigantocellular nucleus, two nuclei important for locomotor function. Using a combination of viral tracing, chemogenetic silencing, and AI-based kinematic analysis, we investigated plasticity and its impact on functional recovery within the first 3 weeks following injury, a period prone to neuronal remodeling. We demonstrate that, in this time frame, while vGlut2-positive fibers within the gigantocellular and lateral paragigantocellular nuclei rewire significantly following cervical spinal cord injury, vGat-positive fibers are rather unresponsive to injury. We also show that the acute silencing of excitatory axonal fibers which rewire in response to lesions of the spinal cord triggers a worsening of the functional recovery. Using kinematic analysis, we also pinpoint the locomotion features associated with the gigantocellular nucleus or lateral paragigantocellular nucleus during functional recovery. Overall, our study increases the understanding of the role of the gigantocellular and lateral paragigantocellular nuclei during functional recovery following spinal cord injury.

## Graphical abstract

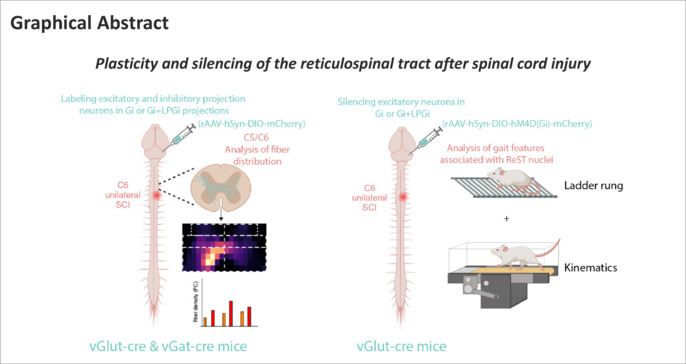

## Introduction

The reticulospinal tract (ReST) is a fundamental neural pathway originating in the brainstem’s reticular formation (RF) and projecting bilaterally down the spinal cord. Its primary role involves the control of posture, balance, and voluntary movements to coordinate complex motor patterns, such as locomotion. The RF, which is spanning the medulla, pons, and mesencephalon is divided into median, medial, and lateral regions with the medial region in the pons and medulla involved in the control of limb movements (Brownstone and Chopek, 2018; Perreault and Giorgi, 2019). The RF is a collection of several nuclei, the most studied nuclei for locomotion being the gigantocellular nucleus (Gi) and the lateral paragigantocellular nucleus (LPGi). One special characteristic of the ReST, in particular of the pontine ReST, is its heterogeneous composition of fibers with about 60% of terminals in the spinal cord positive for vGlut2 and a sizable fraction of terminals positive for vGat (Du Beau et al., 2012; Mitchell et al., 2016).

In the event of a spinal cord injury (SCI), the disruption of functional neural pathways poses significant challenges to motor function. However, the spinal cord exhibits some levels of plasticity, the ability to reorganize and adapt following injury (Bareyre et al., 2004; Filli et al., 2014; Zörner et al., 2014; Bradley et al., 2019). The ReST plays a crucial role in this plasticity, as ReST axons have a high potential for axon growth (Vavrek et al., 2007; Tuszynski and Steward, 2012). This plastic compensation is facilitated through mechanisms like axonal sprouting (Ballermann and Fouad, 2006), synaptic reorganization (Kim et al., 2006), and the formation of new neural circuits that can bypass damaged spinal segments (Filli et al., 2014; May et al., 2017; Mitchell et al., 2016). In particular, increased reticulo-propriospinal contacts paralleled by functional recovery after unilateral cervical hemisection in adult rats indicated that, such as the CST, the ReST can form new circuits to circumvent the lesion (Filli et al., 2014; May et al., 2017). Plasticity of lesioned and unlesioned ReST axons was also reported following incomplete spinal cord lesions (Ballermann and Fouad, 2006; May et al., 2017; Asboth et al., 2018). The fine regulation of the excitation-inhibition (EI) balance in the injured spinal cord circuits is crucial to adaptive plasticity as it allows for the formation of functional and adaptive neural networks while maintaining the stability of the system (Bertels et al., 2022; Punjani et al., 2023). Importantly, the differential contribution of excitatory and inhibitory ReST neurons to this plasticity is currently unknown.

Likewise, while the role of ReST nuclei has progressively started to be unraveled in the mouse (Bouvier et al., 2015; Lemieux and Bretzner, 2019; Cregg et al., 2020; Capelli et al., 2017), the role of the different nuclei of the ReST in the functional recovery of locomotion is still the focus of numerous investigations. Using kinematic testing, it was recently demonstrated that the locally rewired and compensatory Gi plasticity is crucial for the recovery of function and the spontaneous improvement of walking performance after incomplete SCI. It was also estimated that the plasticity of the reticulospinal axons is at least partially causative to the observed functional recovery (Engmann et al., 2020). Recent investigations also examined the contribution of the plasticity of excitatory neurons of the Gi, LPGi, and intermediate reticular nucleus (IRn) to spontaneous motor recovery after SCI (Lemieux et al., 2024). It was shown that photostimulation of LPGi and GiV/α excitatory neurons initiated and speeded locomotion after injury, while photostimulation of Gi excitatory neurons halted locomotion. However, how those specific nuclei control limb movements and specific gait features during recovery is still unclear.

Therefore, in this study, we set out to investigate the plasticity of excitatory vGlut2 and inhibitory vGat neurons located in the Gi and LPGi nuclei of the medullary reticulospinal tract in the context of the functional recovery that follows incomplete cervical lesions of the spinal cord in the first 3 weeks following injury. We also used chemogenetics to silence glutamatergic neurons of the Gi and LPGi and determine how they control specific gait features during functional recovery following spinal cord injury.

## Methods

### Animals

All animal procedures were done according to institutional rules and were approved by the Government of Upper Bavaria on November 2, 2017 (animal protocol Az: 55.2-1-54-2532-61-2017). All experiments were performed in accordance with the German and European regulations. We used mice on a C57Bl/6J background for behavioral experiments not involving silencing. We used VGLUT2-ires-cre-knock-in (C57BL/6J background; Jackson Laboratories, Bar Harbor, ME, USA, RRID: IMSR_JAX:016963) and VGAT-ires-cre-knock-in (C57BL/6J background; Jackson RRID: IMSR_JAX:016962) to perform experiments on excitatory and inhibitory ReST fiber rewiring respectively and for silencing experiments. In this study, mice (100) aged 8–12 weeks at the start of the experiments (starting weight 24.5 ± 0.4 g) and on a mixed gender randomized equally were included. Mice were maintained on a 12‐hour light/ 12‐hour dark cycle with food and water *ad libitum*.

### Plasmid design and virus production

The following plasmids were purchased from Addgene (Watertown, MA, USA): AAV-hSyn-DIO-hM4D(Gi)-mCherry (#44362), pAAV-hSyn-DIO-mCherry (#50459). Adeno-associated viral particles (rAAV) were produced according to a previously described protocol (Challis et al., 2019). HEK293T cells (standard cell line to produce rAAVs; crl3216; ATCC, Teddington, UK) were grown in DMEM (high glucose, GlutaMAX, Thermo Fisher Scientific) supplemented with 10% fetal bovine serum (FBS) and 5% Pen Strep. Cells were transfected using polyethylenimine (PEI) with a 1:4:2 molar ratio of helper, capsid, and desired construct plasmids in DPBS medium without glutamine and serum. Twenty-four hours after transfection, media was changed to serum-supplemented DMEM and the cells were kept for 3 days before harvesting. The supernatant was harvested at 72 and 120 hours after transfection and kept at 4°C. Scraped cells were mixed with SAN + SAN digestion buffer and incubated at 37°C in a water bath for 1 hour. The supernatant was combined with PEG solution overnight and subsequently centrifuged at the speed of 4000 × *g* for 30 minutes at 4°C. The resulting PEG pellet and cell lysate were mixed and loaded on top of an iodoxanol gradient and centrifuged at 350,000 × *g* for 2 hours and 25 minutes at 18°C with slow acceleration. Once the virus was collected from the iodoxanol gradient, it was passed through an Amicon filter and washed multiple times to remove iodoxanol. After the final wash, the virus was resuspended and collected from the filter. Titers were determined using quantitative real-time PCR. Viral aliquots were stored at –80°C until used. Genomic titers were as follows: rAAV2.8-hSyn-DIO-mCherry, 1.1 × 10^11^ genome copies/mL (used to investigate ReST plasticity), and rAAV2.8-hSyn-DIO-hM4D(Gi)-mCherry, 2,1 × 10^12^ GC/mL (used to silence Gi and Gi + LPGi nuclei).

### Surgical procedures

Mice were anesthetized with an intraperitoneal injection using MMF (0.5 mg/kg medetomidine (Orion Pharma, Hambourg, Germany); 5.0 mg/kg midazolam (Ratiopharman, Ulm, Germany) 0.05 mg/kg fentanyl (B. Braun, Melsungen, Germany)) and were given Metacam® (meloxicam; Boeringer Ingelheim, Ingelheim am Rhein, Germany) orally. They were placed on a 38°C heating pad to preserve body temperature before and after surgery. Ophthalmic ointment using Bepanthen was applied to both eyes to prevent desiccation. To ensure adequate anesthesia the pedal reflex was chosen as an indicator of deep pain recognition. Animals have reached the proper plane of anesthesia when the pedal reflex is lost. After surgery, mice were received a subcutaneous injection of an antagonist mixture (atipamezole 2.5 mg/kg, Provident Pharmaceuticals, Athens, Greece; flumazenil 0.5 mg/kg, Hameln Pharma GmbH, Hameln, Germany; and naloxon 1.2 mg/kg, B. Braun). Mice received Metacam® (meloxicam, Boeringer Ingelheim, 1.5 mg/mL) orally for at least 3 days after surgery.

### Spinal cord injury

A laminectomy at cervical level 6 (C6) was performed, followed by a right unilateral dorsoventral lesion of the spinal cord, performed with iridectomy scissors at cervical level 6 (C6), sparing the main component of the corticospinal tract located in the dorsal column. After the lesion was made, the wound was closed, muscles and skin were sutured with surgical staples. In addition, 1 mL of 0.9% NaCl was administered subcutaneously.

### Tracing of the reticulospinal tract from the gigantocellular nucleus or the gigantocellular and lateral paragigantocellular nuclei

For the analysis of ReST rewiring, we used the pAAV2/8-hSyn-DIO-mCherry. An incision is made rostro-caudally across the cranium and Gi injections were made ipsilateral to the lesion with the following coordinates from Bregma according to the Paxinos atlas (Paxinos and Franklin, 2013): rostro–caudal: –6.4 mm, medial–lateral: –0.6 mm, dorso–ventral: –4.2 mm based. To identify plastic changes also occurring from neurons originating in the LPGi, which is a structure difficult to target due to its small size, we targeted the Gi and LPGi and used the following coordinates: rostro-caudal: –6.4 mm, medial–lateral: –1.1 mm, dorso–ventral: –5.9 mm from Bregma. For all injections, we injected 0.3 μL of virus and then the pipette is left in the tissue up to 5 minutes after completion to minimize the backflow and diffusion of the virus.

For acute silencing experiments, VGlut2-ires-cre mice and VGAT-ires-cre mice were injected ipsilateral to the lesion with the pAAV2/8-hSyn-DIO-hM4D(Gi)-mCherry using the same coordinates for the Gi and the Gi + LPGi as above. Clozapine-N-Oxyde (CNO) is used with a concentration of 0.1 mg/kg diluted in saline and was injected at 22 and 43 days following injury at least 45 minutes but not longer than 90 minutes before behavioral readout (Jendryka et al., 2019).

### Behavioral analysis

#### Ladder rung and kinematic analysis

For the ladder rung and kinematic analysis, animals were habituated prior to the start of the experiment and the hindlimb function was evaluated. For the recovery of motor function following spinal cord injury, mice were tested 1 day prior to the lesion and then 4, 21, and 42 days following SCI. For the silencing of ReST nuclei, mice were first habituated and then tested at 21 and 42 days (prior to CNO administration) and 22 and 43 days (acutely after CNO administration). For silencing experiments, all testing was carried out between 45 and 90 minutes post-CNO administration as this has been described as the maximal efficiency (Jendryka et al., 2019). As CNO was reported to affect locomotion at high doses (Chen et al., 2019) we also performed open field behavior to show that at the dose used in our study, CNO did not have any adverse effect on the activity of mice (**Additional Figure 1**). Mice were habituated two times on a horizontal ladder rung with regularly and irregularly spaced rungs and on the treadmill (speed of 15 cm/s). Baseline recordings were performed the day before SCI with the following recordings at 4, 21, and 42 days post-injury (dpi) for the recovery at 21/22 dpi and 42/43 dpi for the silencing. A GoPro 8 camera (GoPro; Munich, Germany; 120 fps) was used to record three runs for each behavioral test. For the ladder rung test, we followed already published protocols (Metz and Whishaw, 2009; Loy et al., 2021) and data were analyzed using BORIS (github.com/olivierfriard/BORIS; Friard and Gamba, 2016). For the kinematic analysis, we used DeepLabCut (github.com/DeepLabCut/DeepLabCut; Mathis et al., 2018) and the ALMA toolbox (github.com/fe1ixxu/ALMA; Aljovic et al., 2022). The parameters used for feature labeling and model training in DeepLabCut were identical to those of previously published work (Aljovic et al., 2022). The ALMA toolbox (Aljovic et al., 2022) was used for the extraction of 44 kinematic parameters. While our injury model produced deficits in both hindlimbs and forelimbs, we focused exclusively on the hindlimbs for our evaluation due to the design of the ALMA toolbox.

#### Open field test

The open field test (Basso et al., 1995) was performed with a video source directly above the apparatus to record the behavior after CNO/saline administration. Testing occurred between 45 and 90 minutes post-administration (Jendryka et al., 2019). Two trials were given and all trials were recorded by the computer-based Any-Maze automated video tracking system (Stoelting Co., Dublin, Ireland).

### Tissue processing and immunohistochemistry

Mice are deeply anesthetized in a closed chamber with isoflurane (AbbVie, North Chicago, IL, USA) until no detectable movement or breathing was observed and perfused with 4% Paraformaldehyde (PFA) in 0.1M phosphate buffer (PB) and the spinal cord was post-fixed for 24 hours in 4% PFA. Tissues were transferred to 30% sucrose solution for two nights before freezing in Tissue-Tek OCT. Spinal cord sections at level C5/C6 above rostral to the lesion and brainstem sections were cut in the cryostat (Leica CM 1850; Leica, Wetzlar, Germany) at 50 μm for analysis of ReST spinal cord rewiring and verifications of brain injections locations. For lesion volume analysis, spinal cord sections were cut at 60 μm. Sections are collected directly onto slides (brain) or free-floating (SC). Sections are mounted in consecutive order to maintain anatomical order and are allowed to dry at room temperature overnight (O/N). For evaluating the accuracy of the injections in the ReST nuclei and the rewiring of Gi and LPGi fibers at cervical level 5/6 (C5/6) we performed immunohistochemistry: briefly, sections were washed three times in 1× PBS for 10 minutes at room temperature and then blocked with 5% horse serum in 0.1% Triton/PBS for 1 hour at room temperature. Then the primary antibody was applied (goat anti-mCherry, 1:500, Cat# AB0081-200 SICGEN, Cantanhede, Coimbra, Portugal) overnight at 4°C. The secondary antibody was then applied (donkey anti-goat Alexa 594, 1:500, Cat# A11058, Thermo Fisher Scientific, Munich, Germany) overnight at 4°C as well as Neurotrace 435/455 (1:500, Cat# N21479, Invitrogen, Waltham, MA, USA) in 1% horse serum and 0.1% Triton/PBS. Sections were then mounted and sealed with Vectashield mounting medium (Cat# H1000, Vector Laboratories, Newark, CA, USA). For the calculation of lesion volume, Neurotrace 435/455 staining (1:500, Cat# N21479, Invitrogen) was performed on the lesioned area (C6).

### Image acquisition

For the brainstem injection analysis, tissue sections were acquired with an upright DM4 B microscope (DM4 B upright fluorescent digital research microscope, Leica) with a QImaging EXI camera at 5× magnification (objective: Leica HC PL FLUOTAR 5×, imaging medium: air, NA: 0.15; 1392 × 1040 pixels, zoom 1×). Tissue sections (3-8 per animal from levels C5–C6) from the spinal cord are scanned with an upright Olympus FV1000 confocal microscope system. All acquisition settings are kept constant between the control and treatment groups for each experiment. To analyze ReST rewiring, confocal mosaic z-stacks are acquired at 20× magnification (objective: Olympus UPLSAPO 20XO, imaging medium: Olympus IMMOIL-F30CC, NA: 0.85; 640 × 640 pixels, zoom 1.1, 0.45 µm z-resolution).

### Quantifications

All quantifications were performed by a blinded observer.

Quantifications of ReST plasticity: To create the heat maps of the cervical (C5–C6) area in Fiji, a custom-written script was used (Loy et al., 2021). We used maximum intensity projections of confocal scans taken with similar scanning settings and aligned them to the central canal for all animals (3–8 sections/animal). Then an average intensity projection was calculated and a heatmap was generated by averaging the intensity within 144 squares over the images (18× 8 square grid). The extracted results for integrated fiber density were corrected for background noise (measured on a grey matter area clearly negative of any fiber) for each section individually using another ImageJ macro (github.com/imagej/ImageJ). The sum of positive integrated density per spinal cord section was normalized to 100%, and then a percentage distribution per square was calculated. Quantifications were then performed over representative areas of the dorsal horn (DH), intermediate laminae (IL) or ventral horn (VH), and subsequent laminae. Laminae were identified using Neurotrace staining. We then calculated a mean percentage of fiber density per area in relation to the entire fiber density in the section. Heatmaps were generated using a self-written Python script that transforms the calculated values per square into color scale across all groups. We then created fold change (FC) heatmaps by dividing each square of the injured map by the mean (per animal) control map. To ascertain that our quantification using heatmaps reflects the real fiber innervation and distribution in the grey matter, we performed a spearman correlation between the collateral length and fiber count evaluated manually in a subset of animals and the density of transduced axons in the spinal cord quantified in the heat maps.

Lesion volume quantification: For calculating the lesion volume, every consecutive section (10 sections per animal) was stained with a fluorescent Nissl dye (NT435; 1:500, N-21479, Thermo Fisher Scientific). Sections were imaged and the lesion area (cavities and surrounding damaged tissue) was outlined and measured in Fiji. For volume calculation, the area was multiplied by the thickness (60 m) between two consecutive sections, and the results were summed.

Brain injection accuracy analysis: For analyzing the accuracy of the viral injections in the desired nuclei, the stained brainstem sections were imaged using a Leica DM4 B upright fluorescent microscope. For Gi injection to be deemed accurate, at least 75% of the total viral spread must be located within the Gi nucleus and less than 25% must be outside of the nucleus with 3% in the LPGi. For Gi+LPGi injections to be deemed accurate at least 75% of the viral spread should be located in the GI and LPGi with at least 30% of the labeling in the LPGi. To illustrate the viral spread for every injection, a custom-made Python script was used. The mCherry signal from the section was converted into a color-coded illustration based on the intensity of the signal.

### Statistical analysis

**Figure 1:**
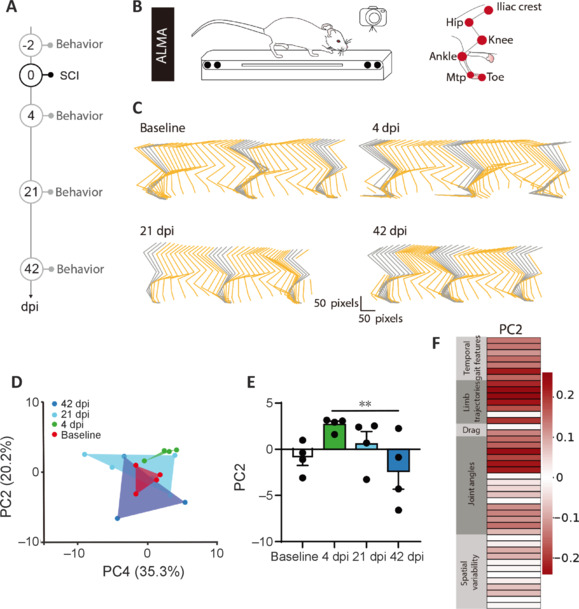
Gait features of locomotion following unilateral spinal cord lesion and over the recovery process. (A) Timeline of the experiment analyzing gait features on the treadmill. (B) Schematic drawing of the treadmill test performed for the ALMA toolbox analysis (left) and markerless paw estimate from Deep Lab Cut (right). (C) Example of a stick plot obtained from the ALMA toolbox to analyze features of gait at baseline, 4, 21, and 42 dpi. (D) Principal component analysis and two-dimensional statistical representation of gait parameters. The area defined by individual points (red: baseline, green: 4 dpi, cyan: 21 dpi, blue: 42 dpi) is traced to show the gait pattern at each time point. (E) Bar graphs of average scores on PC2 (Friedman test and uncorrected Dunn’s *post hoc* test. *P* = 0.055 baseline *vs*. 4 dpi and *P* = 0.006 4 dpi *vs*. 42 dpi). **P < 0.01. (F) Factors loading associated with PC2 indicate that limb trajectories and temporal gait features are responsible for the change of gait at 4 dpi and during the recovery process. *n* = 4. dpi: Day(s) post-injury; PC: principal components; SCI: spinal cord injury.

**Figure 2:**
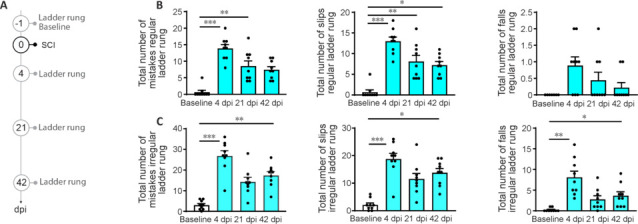
Recovery of rhythmic locomotion and coordinated paw placement in the ladder rung following unilateral lesion of the spinal cord. (A) Timeline of the experiment analyzing behavioral recovery in the ladder rung test. (B) Quantifications of the total number of mistakes (left: Friedman test and Dunn’s multiple comparison test: baseline *vs*. 4 dpi *P* < 0.0001; baseline *vs.* 21 dpi *P* = 0.004), total number of slips (middle: Friedman test and Dunn’s multiple comparison test: baseline *vs.* 4 dpi *P* < 0.0001; baseline *vs*. 21 dpi *P* = 0.008; baseline *vs*. 42 dpi *P* = 0.04), and total number of falls (right: Friedman test and Dunn’s multiple comparison test) in the regular ladder rung test. (C) Quantifications of the total number of mistakes (left: Friedman test and Dunn’s multiple comparison test: baseline *vs*. 4 dpi *P* < 0.0001; baseline *vs*. 42 dpi *P* = 0.005), total number of slips (middle: Friedman test and Dunn’s multiple comparison test: baseline *vs.* 4 dpi *P* < 0.0001; baseline *vs.* 42 dpi *P* = 0.01), and total number of falls (right: Friedman test and Dunn’s multiple comparison test: baseline *vs*. 4 dpi *P* = 0.004; baseline *vs.* 42 dpi *P* = 0.048) in the irregular ladder rung test. *n* = 9. dpi: Day(s) post-injury; SCI: spinal cord injury. *P < 0.05, **P < 0.01, ***P < 0.001.

**Figure 3:**
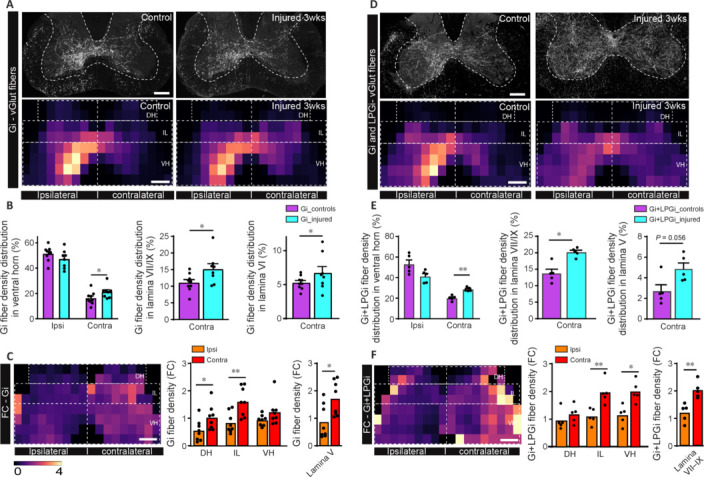
Remodeling of excitatory Gi and Gi+LPGi fibers of the ReST in response to cervical spinal cord injury. (A) Confocal images and heatmaps of overall excitatory ReST fiber density originating from the Gi nucleus in control mice (left) and injured mice (right) at 21 dpi in segment C5/6 above the lesion. (B) Quantification of the density (%) of excitatory ReST fibers originating from the Gi nucleus in the ipsi- and contralateral ventral horn of the cervical spinal cord (left, Mann-Whitney *U* test *P* = 0.047 contralateral ventral horn), in the contralateral lamina VII/IX (middle, Mann-Whitney *U* test *P* = 0.038) and in the contralateral lamina VI (right, Mann-Whitney *U* test *P* = 0.012) in control mice (purple bars) and injured mice (cyan bars) at 21 dpi. (C) Heatmap of FC of excitatory ReST fibers originating from the Gi nucleus between injured and control mice at 21 dpi (left) and quantification of the FC of excitatory ReST fibers originating from the Gi in the ipsilateral and contralateral spinal cord (middle; DH *P* = 0.038; IL *P*=0.007 Mann-Whitney *U* test) and in the contralateral lamina V (right, Mann-Whitney *U* test *P* = 0.038). (D) Confocal images and heatmaps of overall excitatory ReST fiber density originating from the Gi and LPGi nuclei in control mice (left) and injured mice (right) at 21 dpi in segment C5/6 above the lesion. (E) Quantification of the density (%) of excitatory ReST fibers originating from the Gi and LPGi nuclei in the ipsi- and contralateral spinal cord (left, Mann-Whitney *U* test *P* = 0.008 contralateral ventral horn) and in the contralateral lamina VII/IX (middle, Mann-Whitney *U* test *P* = 0.016) and in the contralateral lamina V (right, Mann-Whitney *U* test *P* = 0.056) in control mice (purple bars) and injured mice (cyan bars) at 21 dpi. (F) Heatmap of FC of excitatory ReST fibers originating from the Gi and LPGi nuclei between injured and control mice at 21 dpi (left) and quantification of the FC of excitatory ReST fibers originating from the Gi + LPGi in the ipsilateral and contralateral spinal cord (middle, IL *P* = 0.008; VH *P* = 0.016 Mann-Whitney *U* test) and in the contralateral laminae VII/IX and VI (right, *p* = 0.008 laminae VII/IX Mann-Whitney *U* test). *n* = 5 for Gi + LPGi control and injured groups, *n* = 9 and 8 for Gi control and injured groups respectively. All analysis was carried out in segment C5/6 above the lesion. Scale bars: 200 μm in A, C, D, F. Dotted lines in A, C, D, F indicate the regions of the analysis. Scale bar of the heatmap is indicated in C and also applies for A, D, F. *P < 0.05, **P < 0.01. DH: Dorsal horn; dpi: day(s) post-injury; FC: fold change; Gi: gigantocellular nucleus; IH: intermediate horn; IL: intermediate laminae; LPGi: lateral paragigantocellular nucleus; ReST: reticulospinal tract; VH: ventral horn.

**Figure 4:**
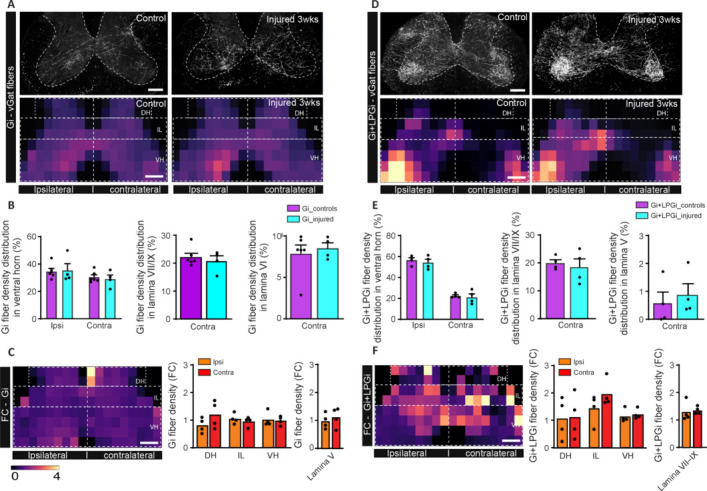
Remodeling of inhibitory Gi and Gi+LPGi fibers of the ReST in response to cervical spinal cord injury. (A) Confocal images and heatmaps of overall inhibitory ReST fiber density originating from the Gi nucleus in control mice (left) and injured mice (right) at 21 dpi in segment C5/6 above the lesion. (B) Quantification of the density (%) of inhibitory ReST fibers originating from the Gi nucleus in the ipsi- and contralateral ventral horn of the cervical spinal cord (left), in the contralateral lamina VII/IX (middle) and in the contralateral lamina VI in control mice (purple bars) and injured mice (cyan bars) at 21 dpi. (C) Heatmap of FC of inhibitory ReST fibers originating from the Gi nucleus between injured and control mice at 21 dpi (left) and quantification of the FC of inhibitory ReST fibers originating from the Gi in the ipsilateral and contralateral spinal cord (middle) and in the contralateral lamina V (right). (D) Confocal images and heatmaps of overall inhibitory ReST fiber density originating from the Gi and LPGi nuclei in control mice (left) and injured mice (right) at 21 dpi in segment C5/6 above the lesion. (E) Quantification of the density (%) of inhibitory ReST fibers originating from the Gi and LPGi nuclei in the ipsi- and contralateral spinal cord (left), in the contralateral lamina VII/IX (middle) and in the contralateral lamina V in control mice (purple bars) and injured mice (cyan bars) at 21 dpi. (F) Heatmap of FC of inhibitory ReST fibers originating from the Gi and LPGi nuclei between injured and control mice at 21 dpi (left) and quantification of the FC of inhibitory ReST fibers originating from the Gi + LPGi in the ipsilateral and contralateral spinal cord (middle) and in the contralateral laminae VII/IX and VI (right). *n* = 4 for Gi + LPGi control and injured groups, *n* = 6 and 4 for Gi control and injured groups respectively. All analysis was carried out in segment C5/6 above the lesion. Scale bars: 200 μm in A, C, D, F. Dotted lines in A, C, D, F indicate the regions of the analysis. Scale bar of the heatmap is indicated in C and also applies for A, D, F. DH: Dorsal horn; dpi: day(s) post-injury; FC: fold change; Gi: gigantocellular nucleus; IH: intermediate horn; IL: intermediate laminae; LPGi: lateral paragigantocellular nucleus; ReST: reticulospinal tract; VH: ventral horn.

**Figure 5:**
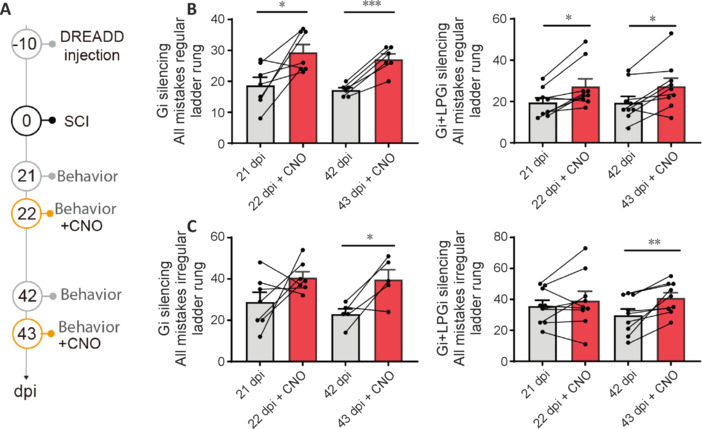
Silencing of Gi or Gi + LPGi ReST nuclei and evaluation of locomotor performance in the ladder rung test. (A) Timeline of the experiment analyzing behavioral recovery in the ladder rung test. (B) Quantifications of the total number of mistakes following silencing of the Gi nucleus (left, Wilcoxon test *P*_21 d_ = 0.031; paired *t*-test *P*_42 d_ = 0.0004) or the Gi + LPGi nuclei (right, paired-test *P*_21 d_ = 0.012; paired *t*-test *P*_42 d_ = 0.01) in the regular ladder rung test. (C) Quantifications of the total number of mistakes following silencing of the Gi nucleus (left, paired *t*-test *P*_42 d_ = 0.034) or the Gi + LPGi nuclei (right, paired *t*-test *P*_42 d_ = 0.006) in the irregular ladder rung test. *n* = 7 for the Gi group; *n* = 9 for the Gi + LPgi group. *P < 0.05, **P < 0.01, ***P < 0.001. dpi: Day(s) post-injury; Gi: gigantocellular nucleus; LPGi: lateral paragigantocellular nucleus; ReST: reticulospinal tract; SCI: spinal cord injury.

**Figure 6:**
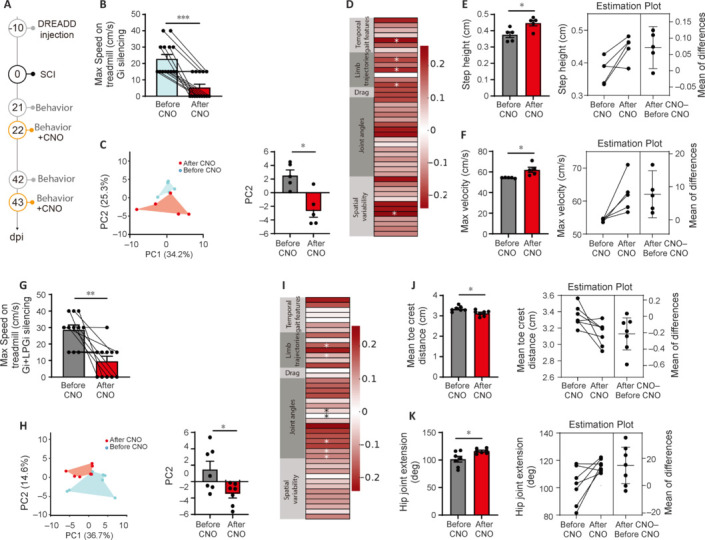
Gait changes in spinal cord injured mice tested on the treadmill using ALMA. (A) Timeline of the silencing experiment following spinal cord injury. (B) Quantification of the maximum speed at which mice can walk on the treadmill before (cyan bar) and after CNO (red bar) in case of Gi silencing (*P* = 0.0005, Wilcoxon matched-pairs signed rank test; *n* = 14). (C) Principal component analysis of data obtained on the treadmill and processed with the ALMA toolbox for spinal cord injury and Gi silencing, and plot of scores of PC2 that represent 25.3% of the variability (principal component analysis, *P* = 0.018, paired *t*-test; *n* = 5). (D) Color-coded representation of factor loadings that identify each parameter’s correlation coefficient (r) with PC2 in case of Gi silencing. Parameters with positive/negative correlation are coded in dark red, while those with a correlation close to 0 are coded in white (see scale on the side). Asterisks indicate significance below 0.05. (E, F) Quantitative evaluation of parameters associated with PC2 in case of Gi silencing, such as step height (E; *P*_step height_ = 0.038, paired *t*-test) and maximum velocity (F; *P*_max velocity_ = 0.04, paired *t*-test). (G) Quantification of the maximum speed at which mice can walk on the treadmill before (cyan bar) and after CNO (red bar) in case of Gi + LPGi silencing (*P* = 0.003, paired *t*-test; *n* = 11). (H) PC analysis of data obtained on the treadmill and processed with the ALMA toolbox for spinal cord injury and Gi + LPGi silencing, and plot of scores of PC2 that represent 14.6% of the variability (principal component analysis, *P* = 0.021, paired *t*-test; *n* = 7). (I) Color-coded representation of factor loadings that identify each parameter’s correlation coefficient (r) with PC2 in case of Gi + LPGi silencing. Parameters with positive/negative correlation are coded in dark red, while those with a correlation close to 0 are coded in white (see scale on the side). Asterisks indicate significance below 0.05. (J, K) Quantitative evaluation of parameters associated with PC2 in case of Gi + LPGi silencing, such as mean toe iliac crest distance (J; *P*_mean toe iliac crest distance_ = 0.036, paired *t*-test) and hip joint extension (K; *P*_hip joint extension_ = 0.036, paired *t*-test). *P < 0.05, **P < 0.01, ***P < 0.001. Gi: Gigantocellular nucleus; LPGi: lateral paragigantocellular nucleus; PC: principal components; SCI: spinal cord injury.

For statistical evaluation, GraphPad Prism 7.01 and 10 (GraphPad Software, San Diego, CA, USA, www.graphpad.com) was used. All results are given as mean ± SEM or as mean with individual data points. Data were tested for normality and based on the results, appropriate testing either parametric or non-parametric was performed. For Figures 1 and 2, we used Friedman test followed by Dunn’s *post hoc* test. For Figures 3 and 4, we used Mann-Whitney *U* tests. For Figures 5 and 6, we used paired Wilcoxon tests and paired *t*-tests. A *P*-value < 0.05 was considered statistically significant.

## Results

### Functional recovery following unilateral lesion of the spinal cord involves recovery of stepping and paw placement

We first investigated the functional deficits and recovery of mice following right unilateral cervical lesion. We observed that mice exhibited bilateral forelimb and hindlimb impairments. In this study, we aimed to determine the functional recovery of the hindlimbs following injury by conducting two types of behavioral experiments. First, we evaluated gait features of hindlimb locomotion on a treadmill using a recently published deep-learning-based toolbox for automated limb motion analysis i.e. the ALMA toolbox (Aljovic et al., 2022). Second, we followed the recovery of rhythmic stepping of the hindlimbs and coordinated hindlimb paw placement using the regular and irregular ladder rung test (Metz and Whishaw, 2009). As behavioral performance can be affected by lesion position and extent, we first verified that the lesion was reproducible in a subset of animals. Indeed, quantification of lesion volume did not show strong variability between animals (183 ± 10 mm^3^, **Additional Figure 2**). We then investigated which gait features are affected by the injury and the recovery process using the ALMA toolbox for analysis of kinematic parameters (Aljovic et al., 2022). To do so, we used the animals whose injury level allowed them to walk on the treadmill 4 days post-injury. We trained mice (*n* = 4) on a treadmill before SCI (15 cm/s) and tested them and recorded their gait at baseline and 4, 21, and 42 days post-injury (**Figure 1A** and **B**). We used the ALMA toolbox to extract 44 features of gait and performed a principal component analysis (PCA) to reduce dimensionality and provide a comprehensive quantification of the locomotor features (**Figure 1C** and **D**). We visualized gait patterns in 2-D coordinates with PC1 and PC2 explaining the highest variance (35.3% and 20.2% respectively) and determined that PC2 best captured the injury features (**Figure 1D**). In PC2, we observed that the scores at 42 dpi were significantly different from those at 4 dpi (**Figure 1E**). The distinct characteristics associated with PC2 were obtained from the analysis of factor loadings and grouped into clusters according to the ALMA toolbox (**Figure 1F** and **Additional Figure 3**). Factor loading revealed that several parameters related to limb trajectories and joint angles were highly correlated to PC2 and explained the recovery profile of animals following unilateral spinal cord lesions (**Figure 1F** and **Additional Table 1**).

**Additional Table 1:** Description of parameters calculated by the ALMA toolbox

Parameter clusters	Parameters
**Temporal features of gait**	Stance duration (s) Swing duration (s) Swing percentage (%) Stance percentage (%) Max velocity during swing (cm/s) Cycle duration (s) Cycles duration (# frames)
**Limb endpoint trajectories**	Cycle velocity (cm/s) Stride length (cm) Mean toe-to-crest distance (cm) Max toe-to-crest distance (cm) Min toe-to-crest distance (cm) Toe-to-crest distance SD (cm) Step height (cm)
**Drag**	Drag duration (s) Drag percentage (%)
**Joint angles**	Mtp joint extention (deg) Mtp joint flexion (deg) Mtp joint amplitude (deg) Mtp joint SD (deg) Ankle joint extention (deg) Ankle joint flexion (deg) Ankle joint amplitude (deg) Ankle joint SD (deg) Knee joint extention (deg) Knee joint flexion (deg) Knee joint amplitude (deg) Knee joint SD (deg) Hip joint extention (deg) Hip joint flexion (deg) Hip joint amplitude (deg) Hip joint SD (deg)
**Spatial variability**	DTW distance x plane 5 stride mean DTW distance x plane 5 stride SD DTW distance y plane 5 stride mean DTW distance y plane 5 stride SD DTW distance xy plane 5 stride mean DTW distance xy plane 5 stride SD DTW distance x plane 10 stride mean DTW distance x plane 10 stride SD DTW distance y plane 10 stride mean DTW distance y plane 10 stride SD DTW distance xy plane 10 stride mean DTW distance xy plane 10 stride SD

DTW: Dynamic time warping; Mtp: metatarsophalangeal; SD: standard deviation.

Then, we investigated the recovery of rhythmic stepping using the ladder rung test (Metz and Whishaw, 2009). We observed that mice (*n* = 9) who sustained unilateral lesions of the spinal cord and were tested at 4, 21, and 42 days following lesion (**Figure 2A**) showed a strong bilateral hindlimb impairment illustrated by an increase of total mistake in the regular and irregular ladder rung test that recovered partially overtime (**Figure 2B** and **C**). When we separated the slips (slipping from the rung while maintaining the body posture) from the true falls (missing the rung altogether), we noticed that in the regular ladder rung, almost all mistakes were slips while in the irregular ladder rung both mistakes occurred frequently and were demonstrating a similar recovery pattern (**Figure 2B** and **C**). This illustrates that both rhythmic stepping and coordinated paw placement are impaired following unilateral lesion of the spinal cord and recover over time.

### Functional recovery is paralleled by specific remodeling of excitatory reticulospinal fibers

The reticulospinal tract is not only important to control locomotion in healthy mice but also following spinal cord injury as it allows the re-establishment of walking (Filli et al., 2014; Zörner et al., 2014; Engmann et al., 2020). To determine the remodeling of excitatory and inhibitory reticulospinal fiber within the Gi and LPGi nuclei, we used respectively vGlut2-cre and vGat-cre mice. We labeled these mice with cre-dependent AAVs expressing mCherry injected either in the gigantocellular nucleus (Gi; vGlut: *n* = 9 controls and *n* = 8 injured; vGat: *n* = 6 controls and *n* = 4 injured) or in the gigantocellular and the lateral paragigantocellular nucleus (Gi + LPGi; vGlut *n* = 5 controls and injured; vGat *n* = 4 controls and injured) ipsilateral to the lesion. We chose to analyze LPGi plasticity together with the Gi due to the difficulty of targeting the LPGi alone as it is a very small nucleus. We first verified the accuracy of all our injections (**Additional Figure 4**) before analyzing the distribution of the fibers originating from the Gi or the Gi + LPGi in the cervical spinal cord (segment C5/6 above the lesion). To determine whether excitatory and inhibitory fibers originating from the Gi and the Gi + LPGi remodel following unilateral spinal cord lesion, we analyzed the dense topographical innervation and distribution of fibers in the cervical segments C5–C6 above the lesion using heatmaps of the axonal grey matter expression in vGlut2-cre and vGat-cre mice. We first verified that our heatmaps analysis reflects the true fiber innervation and distribution in the grey matter by performing correlation of the collateral length and fiber count to the density of transduced axons in the spinal cord quantified in the heat maps (**Additional Figure 5**). We then proceeded to map regional densities of either excitatory or inhibitory fibers coming from the Gi or the Gi + LPGi 21 days following cervical spinal cord injury and represented them as heatmaps (as described above; **Figure 3** for excitatory profiles and **Figure 4** for inhibitory profiles). Here we selected 6 regions of interest: dorsal horn, intermediate laminae and ventral horn ipsi- and contra-lateral to the lesion (**Figures 3** and **4**). For excitatory fibers, we mapped first the regional densities from injection in the Gi in vGlut2-cre mice (**Figure 3A**). We found that following the spinal cord injury the excitatory Gi contralateral innervation of the spinal cord was slightly, albeit significantly, increased in the ventral horn at 21 days post-injury and that this increase was specifically significant in laminae VI and VII–IX (**Figure 3B**). Interestingly, the ipsilateral excitatory innervation from the Gi levels was not significantly changed (**Figure 3B**) and the contralateral innervation in the dorsal and intermediate horns was also not significantly affected (**Additional Figure 6A**). The fold change heatmap of the Gi when injured and control mice were compared demonstrated a slight but significant general increase in density between the ipsilateral and contralateral sides in the dorsal horn and intermediate lamina, and a significant regional increase in lamina V was found (**Figure 3C**). This underscores that subtle plastic changes of Gi excitatory fibers mostly occur in the contralateral ventral horn 21 days post-injury. We then focused on the injections that targeted not only the Gi but also the LPGi to determine whether the additional labeling of the lateral paragigantocellular nucleus would reveal additional plasticity (**Figure 3D**).

Labeling of both Gi and LPGi nuclei demonstrate that there is an overall decrease in ipsilateral innervation balanced by an increase in contralateral innervation (**Figure 3D** and **E**). By concentrating on the contralateral side, we noticed that the increase was particularly significant in laminae V and VII–IX (**Figure 3E**) which is reminiscent of the plasticity seen when the Gi alone was labeled although it appears increased here. Similarly, to the Gi labeling, we did not notice any significant changes in ipsilateral and contralateral innervation of the dorsal and intermediate horns (**Additional Figure 5B**). The fold change heatmap of the Gi + LPGi injection demonstrated significantly more differences between the ipsilateral and contralateral sides with a significant increase in the intermediate and ventral horns that was particularly noticeable in laminae VII–IX (**Figure 3F**).

We then turned our attention to inhibitory fibers located in the Gi and LPGi. To label those fibers we used vGat-cre mice (**Figure 4A**) and we either labeled the fibers originating from the Gi (**Figure 4A–C** and **Additional Figure 5C**) or the Gi and LPGi (**Figure 4D–F** and **Additional Figure 5D**). In contrast to excitatory fibers, we could not detect any changes in heatmaps neither between control and injured mice nor between the ipsilateral and contralateral spinal cord sides for any injections considered. This suggests that unlike excitatory fibers, inhibitory fibers from the Gi and LPGi do not remodel following spinal cord injury (**Figure 4**). Finally, to ascertain that vGlut and vGat neurons behave differently in terms of their plastic abilities following SCI and that the lack of significance in the vGat group is due to a lack of plasticity, we compared the fold changes of vGlut and vGat fibers to each other in the previously defined regions (**Additional Figure 7**). In accordance with our previous data showing no changes in distribution in the ipsilateral spinal cord, we did not see any significance ispilaterally (**Additional Figure 7A** and **B**). In the contralateral hemisphere, we could detect significance in the Gi and Gi-LPGi in particular in the intermediate laminae and ventral horn (specifically also lamina VII/IX) respectively (**Additional Figure 7C** and **D**) suggesting that vGlut and vGat neurons differ indeed in their post-injury plastic abilities.

### Silencing of excitatory fibers from the gigantocellular and lateral paragigantocellular nuclei decreases locomotor performance and reveals their importance for specific gait features

As excitatory fibers are the only subpopulation showing plasticity following unilateral spinal cord lesions, we then set to silence those fibers in specific nuclei. To do so, we took advantage of chemogenetics which allows the specific and timely silencing of subsets of neurons. We use the AAV-DIO-hM4Di-mCherry in combination to Clozapine-N-Oxyde (CNO) in vGlut2-cre mice to silence excitatory neurons in the ReST nuclei ipsilesionally and evaluated functional recovery in the regular and irregular ladder rung test (**Figure 5A–C**). We first investigated whether CNO alone would have off-target effects and would affect the behavior in the open field at the dose administered. We could not see any differences in mice (*n* = 4 per group) after administration of saline or CNO (**Additional Figure 1**). We found that silencing of the Gi (*n* = 7) or the Gi + LPGi (*n* = 9) triggers a profound loss of the recovered function both in the regular and irregular ladder rung at the two time points at which the silencing was performed e.g. 22 and 43 days following the injury (**Figure 5B** and **C**). Specifically, mice performed worse in the regular ladder rung following Gi silencing at 21 days (18.7 ± 2.6 mistakes before *vs.* 29.4 ± 2.5 mistakes after; *P* = 0.0312) and 42 days (17.2 ± 0.8 mistakes before *vs*. 27.2 ± 1.7 mistakes after; *P* = 0.0004). Similar results were found for silencing of the Gi + LPGi silencing at 21 days (19.6 ± 2.2 mistakes before *vs.* 27.3 ± 3.6 mistakes after; *P* = 0.0115 and 42 days (19.4 ± 3.0 mistakes before *vs*. 27.4 ± 3.9 mistakes after, *P* = 0.0104). Interestingly, in the irregular ladder rung, no significant worsening could be seen at 21 days for the Gi (28.9 ± 4.7 mistakes before *vs*. 40.7 ± 2.8 mistakes after) or Gi + LPGi silencing (35.7 ± 3.8 mistakes before *vs*. 39.2 ± 6.0 mistakes after respectively). However, at 42 days post-injury we could detect a worsening of the performance in the irregular ladder rung for the Gi (23.0 ± 2.5 mistakes before *vs*. 39.8 ± 4.7 mistakes after, *P* = 0.0336) and Gi + LPGi (29.8 ± 4.0 mistakes before *vs*. 41.0 ± 3.3 mistakes after, *P* = 0.0063) silencing.

We then performed gait analysis using our ALMA toolbox to determine which gait features are affected by silencing of specific ReST nuclei (**Figure 6A–K**). To do so, mice (*n* = 17 per group) walking on a treadmill at 15 cm/s were recorded at 21 and 42 days post-injury before and after acute silencing using chemogenetics (**Figure 6A**). We first tested their ability to walk on the treadmill before and after silencing. We found that the motor performance in the treadmill decreases from 22.9 ± 2.7 to 5.4 ± 2.0 cm/s for the Gi silencing (*P* = 0.0005) and from 28.6 ± 2.9 to 9.5 ± 3.0 cm/s for the Gi + LPGi silencing (*P* = 0.003) indicating that recovered locomotion was strongly perturbed by silencing of either of the nuclei (**Figure 6B** and **G**). We then performed kinematic analysis on the mice that could walk at 15 cm/s on the treadmill before and after silencing (*n* = 5 for the Gi and *n* = 7 for the Gi + LPGi silencing) and compared their gait features using principal component analysis to determine which parameters are dependent on the Gi or the LPGi during recovery of function. PC1 and PC2 together represented 59.3% of the variance for the Gi silencing and 51.3% of the variance for the Gi + LPGi silencing with PC2 better reflecting the between-group differences in both cases (*P*_Gi_ = 0.0176; *P*_Gi + LPGi_ = 0.0209; **Figure 6C** and **H**). After calculating the factor loadings, we identified the parameters that best-represented PC2 in the case of Gi silencing. Those parameters are mostly related to limb trajectories (**Figure 6D** and **Additional Figure 8A**) such as, among others, step height or maximum velocity that demonstrated significant modifications after CNO injections (*P*_step height_ = 0.0375; *P*_max velocity_ = 0.0395; **Figure 6E** and **F**). Interestingly the silencing of the Gi + LPGi allowed us to indirectly tease out the association of the LPGi with some of the gait parameters. When we observe the factor loadings to identify parameters associated with PC2 in the case of Gi+LPGi silencing we find that in addition to limb trajectories parameters, joint angles parameters are strongly changing in association with PC2 during silencing of the recovered function (**Figure 6I** and **Additional Figure 8B**). In particular, parameters such as toe-iliac crest distance and hip joint extension were shown to be influenced by the silencing (*P*_mean toe iliac crest distance_ = 0.0364; *P*_hip joint extension_ = 0.0355; **Figure 6J** and **K**). These data suggest an association of gait parameters related to limb trajectories with the Gi and gait parameters related to joint angles with the LPGi during recovery of function following SCI.

## Discussion

In this study, we set out to determine the plastic capacities of several nuclei of the ReST with an emphasis on understanding the differences between the plastic abilities of excitatory vGlut2 and inhibitory vGat fibers. To unravel the role of those fibers in the recovery process and in particular their involvement with particular gait features, we then silenced the fibers undergoing plasticity changes following SCI to determine the role of important ReST nuclei to the recovery following spinal cord injury in mice.

### Functional recovery after specific reticulospinal tract is paralleled by the plasticity of excitatory fibers

The first aim of this study was to untangle the role of excitatory and inhibitory fibers with respect to their plastic abilities above the lesion site following spinal cord injury. Here we find significant neuroplasticity and adaptations of excitatory fibers above the lesion site following injury. Such adaptations rostral to the lesions were previously reported for the ReST without any distinction of neurotransmitter profile (Filli et al., 2014; Engmann et al., 2020). We describe plasticity with increased sprouting in the contralateral hemisphere in the case of the Gi and LPGi + Gi labeling suggesting compensatory plasticity in response to the injury. The ReST is an interesting tract as it contains both excitatory vGlut2 and inhibitory vGat fibers (Vetrivelan et al., 2009; Martin et al., 2011; Du Beau et al., 2012; Hossaini et al., 2012; Mitchell et al., 2016). In our study that focused on the medullary ReST, we could find a large number of excitatory and a smaller fraction of inhibitory fibers running together in the lateral funiculus. This is in agreement with the above-mentioned studies on the pontine ReST. To our knowledge, the differential plasticity of excitatory vs inhibitory descending motor fibers of the ReST has not yet been examined. However, it is noteworthy that functionally adaptive plasticity involves a refined balance between the plasticity of excitatory and inhibitory fibers (Asboth et al., 2018; Wu et al., 2022). While excitatory fibers allow the establishment and the strengthening of new synaptic connections (Filli et al., 2014; Asboth et al., 2018; Bradley et al., 2019; Van Steenbergen et al., 2023) thereby allowing functional recovery, a fine balance is necessary to ensure the refinement and stabilization of the new connections and allows for the stability of the system (Bannon et al., 2020). The fact that we could not see any rewiring or sprouting of inhibitory fibers following SCI in contrast to excitatory fibers might suggest an imbalance between excitatory and inhibitory drive. Interestingly, changes in the excitatory-inhibitory ratio have been reported in the cortex following stroke in animals and humans (Clarkson et al., 2010; McDonnell and Stinear 2017; Huang et al., 2018) with a decreased GABAergic inhibition in the ipsilesional hemisphere. There, it is suggested that decreased inhibition in early stroke may play an important role in supporting neuroplasticity and functional recovery. In the chronic phase after stroke, as inhibition normalizes, the potential for spontaneous motor recovery decreases (Grigoras and Stagg, 2021). We believe that the imbalance in excitatory-inhibitory plasticity in the early phase following spinal cord injury that favors excitation is likely to be crucial for functional recovery too and might highlight the plasticity window during which functional recovery is achievable (Grigoras and Stagg, 2021; Joy and Carmichael, 2021). In support of these speculations are rehabilitation studies in stroke patients that demonstrate that functional recovery driven by rehabilitation also lowers inhibition (Blicher et al., 2015; Ferreiro de Andrade and Conforto, 2018). Following spinal cord injury, the excitatory-inhibitory ratio is also important to command locomotor recovery. For example, in the zebrafish, complete spinal cord injury is followed by the regrowth of a large majority of excitatory interneurons so that the ratio of excitatory-inhibitory interneurons increases from 2 (before the injury) to 10 fold after the injury (Huang et al., 2021). These excitatory neurons reconnect the separated spinal cord segments and provide excitatory input important for functional recovery. Additional work demonstrates that following complete spinal cord injury in adult mice, there is a neurotransmitter switching of specific excitatory interneurons to an inhibitory phenotype promoting inhibition on motor neurons. Using genetic manipulations to induce this inhibitory switch in neonatal mice, abolishes functional recovery (Bertels et al., 2022). Interestingly, it was also reported that epidural stimulation during locomotor training activates both excitatory and inhibitory spinal interneurons which likely contributes to the complex functional recovery observed during and after epidural stimulation (Skinnider et al., 2021). Finally, while no structural changes of inhibitory input from the Gi/LPGi could be observed in this study, adaptations in inhibitory synaptic strength could likely contribute to functional recovery as changes in inhibitory synaptic signaling is an adaptive feature following spinal cord injury (Bras and Liabeuf, 2021). In particular, it is recognized that inhibitory input can decrease the excitability of monosynaptic reflex to afferent inputs and improve spasticity and locomotion following SCI (Hofstoetter et al., 2020).

### Specific silencing of reticulospinal tract nuclei identifies gait parameters involved in several aspects of locomotion during functional recovery

We then set out to understand the functional role of excitatory fibers as the sole actors of the rewiring process following SCI. To do so, we used chemogenetics to silence given subpopulations of excitatory fibers either originating from the Gi nucleus or the Gi and LPGi nuclei. We focused on the Gi and LPGi as those two nuclei have been reported to be two fundamental nuclei for locomotion (Capelli et al., 2017; Lemieux and Brezner, 2019; Cregg et al., 2020; Usseglio et al., 2020; Lemieux et al., 2024). We used chemogenetics as this is a versatile method to manipulate activity with spatial and temporal resolution and without the invasiveness of the ligand administration (Van Steenbergen and Bareyre, 2021). We did not see any off-target effects of CNO at the doses that we used in this study which validated our approach for behavioral evaluation. This is important to point out as some studies have reported that CNO alone at doses higher than the one we used in this study can alter startle reflex or even locomotion (MacLaren et al., 2016). Here we used doses well below the doses describing those effects and did not see any effect on locomotion when using the open field test. Using the ladder rung test, we could see that acute silencing of the remodeled excitatory fibers in the Gi or LPGi induces a decrease in locomotor performance both in the regular ladder rung important for rhythmic lomocotion and in the irregular ladder rung that tests fine paw placement. This confirms several studies that have demonstrated the importance of the Gi and LPGi for locomotor recovery after SCI (Caggiano et al., 2018; Engmann et al., 2020). Here we locate this feature in the remodeling of excitatory fibers which confirms the findings of a recent study (Lemieux et al., 2024). Interestingly the performance in the regular ladder rung test was more affected at 21 days than the performance in the irregular ladder rung that was only perturbed at 42 days. This suggests that silencing reticulospinal neurons of the Gi or LPGi + Gi impairs more a stereotypic locomotion than a more skilled locomotor task, at least at 21 days following injury. This might be related to the gait features primarily affected by the Gi and LPGi. To determine which features of gait are dependent on the Gi and LPGi nuclei, we performed gait analysis. Interestingly, we could reveal that most gait features affected by the silencing of excitatory fibers originating from the Gi are responsible for parameters associated with limb endpoint trajectories such as step height, stride length, toe-iliac crest distance or cycle velocity while most gait features associated with the Gi + LPGi are concerning limb endpoint trajectories and joint angles in particular hip, knee, ankle and metatarsophalangeal joint angles, flexion and extension. While the behavior associated with excitatory neurons of the Gi and LPGi were reported before, with the LPGi important to initiate and accelerate locomotion after SCI and the Gi important to stop locomotion (Lemieux et al. 2024), the gait features controlled by glutamatergic neurons in the Gi and LPGi were not reported. Here we now add to the knowledge on the plasticity of those nuclei following spinal cord injury and demonstrate that each of them is responsible for specific gait features during recovery. Finally, as gait features associated with those nuclei start to be unraveled and as axonal rewiring is often supported by rehabilitative strategies such as intensive physical therapy, our work now opens the way to neuromodulatory techniques to enhance specific gait features and promotes adaptive locomotor recovery by fine-tuning the excitatory-inhibitory balance within the injured spinal cord.

### Limitations

There are several limitations to our study. First, because all ReST nuclei, in particular the very small and restricted LPGi, are very difficult to access and selectively manipulate, we could only outline the role of the LPGi in plasticity and in gait features indirectly by differentially labeling the LPGi with the Gi and comparing to the Gi alone. Second, despite our careful verifications of the injection sites, it cannot be excluded that other nuclei surrounding the Gi and LPGi could have been labeled “en passant” therefore altering the plasticity and silencing experiments. Due to the limited number of time points investigated in the present study for ReST plasticity, it also cannot be excluded that the timeline of inhibitory remodeling could be shifted to later time points in comparison to excitatory plasticity thereby only creating a temporary imbalance in input. Third, we did not quantify the density of synaptic terminals. Therefore, the increase in axonal sprouting of vGlut2 fibers following SCI might not be reflecting an increased number of synapses. Finally, the absence of baseline before SCI in our silencing experiments could be a confounder to our conclusions.

## Conclusion

We have shown in this study that while excitatory fibers from the ReST within the Gi and LPGi nuclei rewire significantly following cervical lesions of the spinal cord, inhibitory fibers do not. Using specific acute silencing of the rewiring excitatory ReST fibers, we have also shown a worsening of the functional recovery that occurs following incomplete injuries and using detailed kinematic analysis, we have identified the gait features associated with the Gi or LPGi nuclei during functional recovery. Overall, our study increases our understanding of (i) the post-injury rewiring capacities of diverse components of the ReST and (ii) the role of the Gi and LPGi nuclei during recovery of locomotion following SCI.

## Additional files:

***Additional Figure 1:*** *Distance travelled by mice receiving saline or CNO in absence of DREADDs.*

Additional Figure 1 Distance travelled by mice receiving saline or CNO in absence of DREADDs.
(A) Schematic drawing of the distance travelled by mice in the open field. (B) Quantification (m) of the distance traveled in the open field after saline (white bar) or CNO administration (red bar). CNO: Clozapine-N-Oxyde.

***Additional Figure 2:*** *Quantification of unilateral lesion volume.*

Additional Figure 2 Quantification of unilateral lesion volume.
(A) Confocal micrograph of unilateral spinal cord lesion stained with neurotrace 435 (NT435: cyan). (B) Quantification of lesion volume in vGlut2-cre mice injected in the Gi + LPGi (n=9) or in the Gi (n=8). No differences in lesion volume. Scale bar: 500μm. Dashed lines outline the lesion. Gi: Gigantocellular nucleus; LPGi: lateral paragigantocellular nucleus.

***Additional Figure 3:*** *Detailed representation of PC1 and PC2 factor loadings during recovery following spinal cord injury.*

Additional Figure 3 Detailed representation of PC1 and PC2 factor loadings during recovery following spinal cord injury.
Color-coded representation of factor loadings that identify each parameter's correlation coefficient (r, written in each line) with PC1 and PC2 during recovery following spinal cord injury. Parameters with positive/negative correlation are coded in dark red, while those with a correlation close to 0 are coded in white (see scale on the side). DTW: Dynamic time warping: SD: standard deviation.

***Additional Figure 4:*** *Representation fluorescent images of typical Gi and Gi+LPGi injections.*

Additional Figure 4 Representative fluorescent images of typical Gi and Gi + LPGi injections.
(A) Representation fluorescent images of typical Gi injections. (B) Representation fluorescent images of typical Gi + LPGi injections. Scale bars equal 500μm.; Gi: Gigantocellular nucleus; LPGi: lateral paragigantocellular nucleus.

***Additional Figure 5:*** *Correlation of heatmaps quantifications with manual evaluation of collateral length and fiber count.*

Additional Figure 5 Correlation of heatmaps quantifications with manual evaluation of collateral length and fiber count.
(A) Correlation of the grey matter viral density evaluated to generate the heat maps with the manual collateral length. Spearman correlation coefficient (r) is presented as well as the unparametric p value of the correlation. (B) Correlation of the grey matter viral density evaluated to generate the heat maps with the manual fiber count. Spearman correlation coefficient (r) is presented as well as the unparametric p value of the correlation.

***Additional Figure 6:*** *Remodeling of excitatory and inhibitory ReST Gi and Gi+LPGi fibers in response to cervical spinal cord injury in the intermediate and dorsal horn.*

Additional Figure 6 Remodeling of excitatory and inhibitory ReST Gi and Gi + LPGi fibers in response to cervical spinal cord injury in the intermediate laminae and dorsal horn.
(A) Quantification of the intensity of excitatory ReST fibers originating from the Gi nucleus in the ipsi and contralateral intermediate horn and dorsal horn of the cervical spinal cord in control mice (purple bars) and injured mice (cyan bars) at 21 dpi. (B) Quantification of the intensity of excitatory ReST fibers originating from the Gi+LPGi nucleus in the ipsilateral (ipsi) and contralateral (contra) intermediate horn and dorsal horn of the cervical spinal cord in control mice (purple bars) and injured mice (cyan bars) at 21 dpi. (C) Quantification of the intensity of inhibitory ReST fibers originating from the Gi nucleus in the ipsi and contralateral intermediate horn and dorsal horn of the cervical spinal cord in control mice (purple bars) and injured mice (cyan bars) at 21 dpi. (D) Quantification of the intensity of inhibitory ReST fibers originating from the Gi + LPGi nucleus in the ipsi and contralateral intermediate horn and dorsal horn of the cervical spinal cord in control mice (purple bars) and injured mice (cyan bars) at 21 dpi. dpi: days post-injury; Gi: gigantocellular nucleus; LPGi: lateral paragigantocellular nucleus; ReST: reticulospinal tract.

***Additional Figure 7:*** *Quantification of the FC of excitatory (vGlut2) and inhibitory (vGat) fibers following spinal cord injury ipsilateral and contralateral to the lesion.*

Additional Figure 7 Quantification of the FC of excitatory (vGlut2) and inhibitory (vGat) fibers following spinal cord injury ipsilateral and contralateral to the lesion.
(A) Quantification of the FC of excitatory (vGlut2) and inhibitory (vGat) fibers ipsilateral following Gi injections. (B) Quantification of the FC of excitatory (vGlut2) and inhibitory (vGat) fibers ipsilateral following Gi + LPGi injections. (C) Quantification of the FC of excitatory (vGlut2) and inhibitory (vGat) fibers contralateral following Gi injections. P = 0.049 in the intermediate lamina (IL; Mann-Whitney *U* test) (D) Quantification of the FC of excitatory (vGlut2) and inhibitory (vGat) fibers contralateral following Gi + LPGi injections. *P* = 0.016 in the VH and laminae VII-IX (Mann-Whitney *U* test). DH: Dorsal horn; FC: fold change; Gi: gigantocellular nucleus; IL: intermediate laminae; LPGi: lateral paragigantocellular nucleus; vGat: GABAergic; vGlut2: glutamatergic; VH: ventral horn.

***Additional Figure 8:*** *Detailed representation of PC2 factor loadings for Gi and LPGi silencing during gait analysis.*

Additional Figure 8 Detailed representation of PC2 factor loadings for Gi and LPGi silencing during gait analysis.
(A) Color-coded representation of factor loadings that identify each parameter's correlation coefficient (r, written in each lines) with PC2 in case of Gi silencing. Parameters with positive/negative correlation are coded in dark red, while those with a correlation close to 0 are coded in white (see scale on the side). *P* values are in brackets. (B) Color-coded representation of factor loadings that identify each parameter's correlation coefficient (*r*, written in each lines) with PC2 in case of Gi + LPGi silencing. Parameters with positive/negative correlation are coded in dark red, while those with a correlation close to 0 are coded in white (see scale on the side). *P* values are in brackets. Gi: Gigantocellular nucleus; LPGi: lateral paragigantocellular nucleus; PC: principal components.

***Additional Table 1:*** *Description for parameters calculated by the ALMA toolbox.*

## Data Availability

*All relevant data are within the paper and its Additional files*.
