# Supplementary figures and images for: Differential plasticity of excitatory and inhibitory reticulospinal fibers after spinal cord injury: Implication for recovery
Source: Neural Regen Res. 2025 Feb 24;21(5):2011–20. doi: 10.4103/NRR.NRR-D-24-01060 (PMC12694638; doi:10.4103/NRR.NRR-D-24-01060)

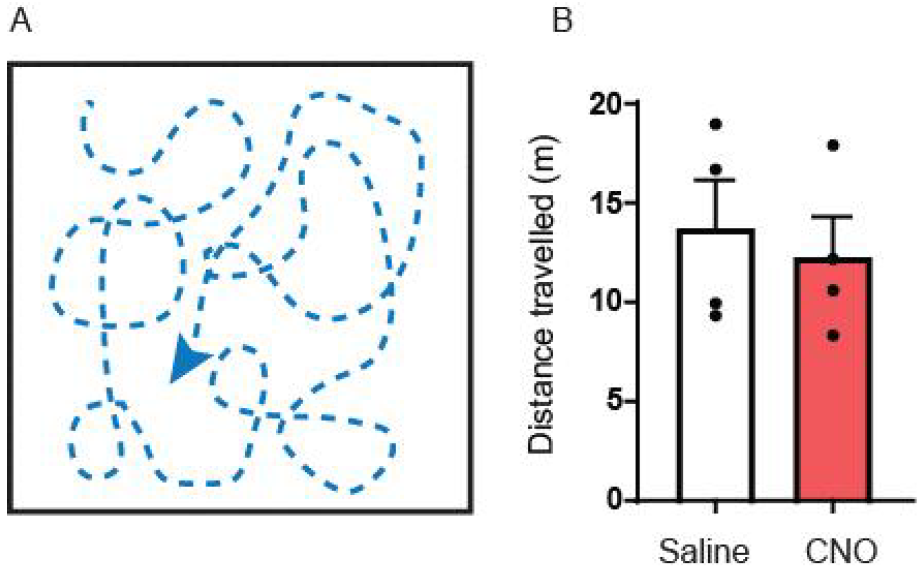

Supplement: Supplementary file 1 [file NRR-21-2011_Suppl1.tif]

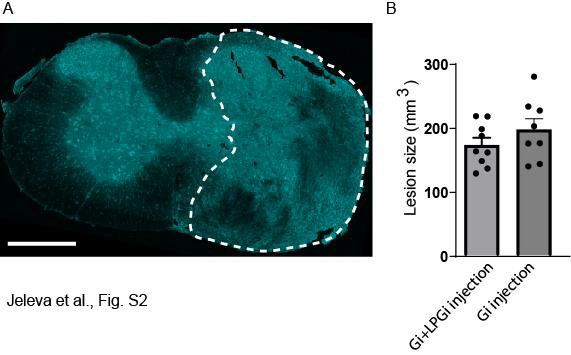

Supplement: Supplementary file 2 [file NRR-21-2011_Suppl2.tif]

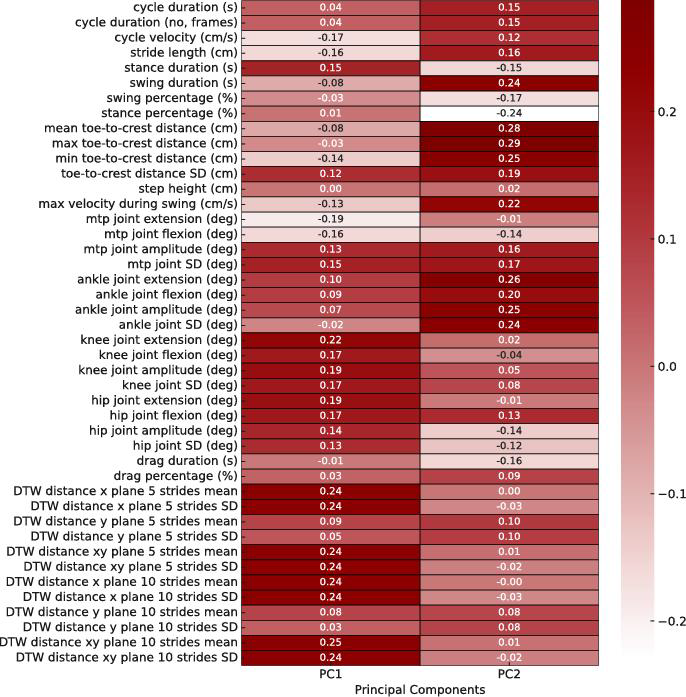

Supplement: Supplementary file 3 [file NRR-21-2011_Suppl3.tif]

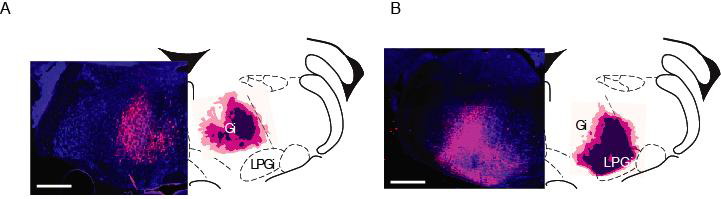

Supplement: Supplementary file 4 [file NRR-21-2011_Suppl4.tif]

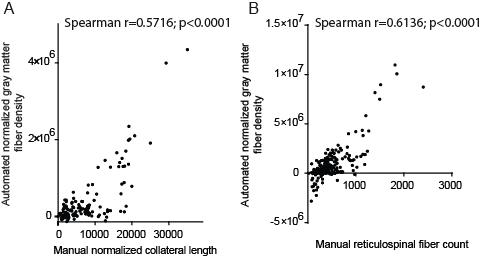

Supplement: Supplementary file 5 [file NRR-21-2011_Suppl5.tif]

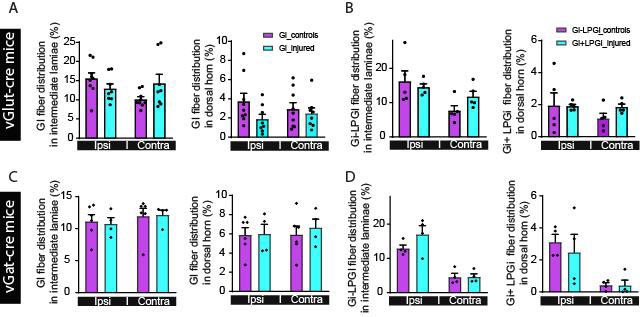

Supplement: Supplementary file 6 [file NRR-21-2011_Suppl6.tif]

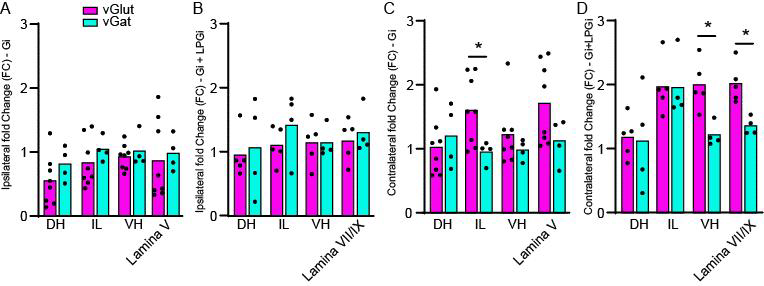

Supplement: Supplementary file 7 [file NRR-21-2011_Suppl7.tif]

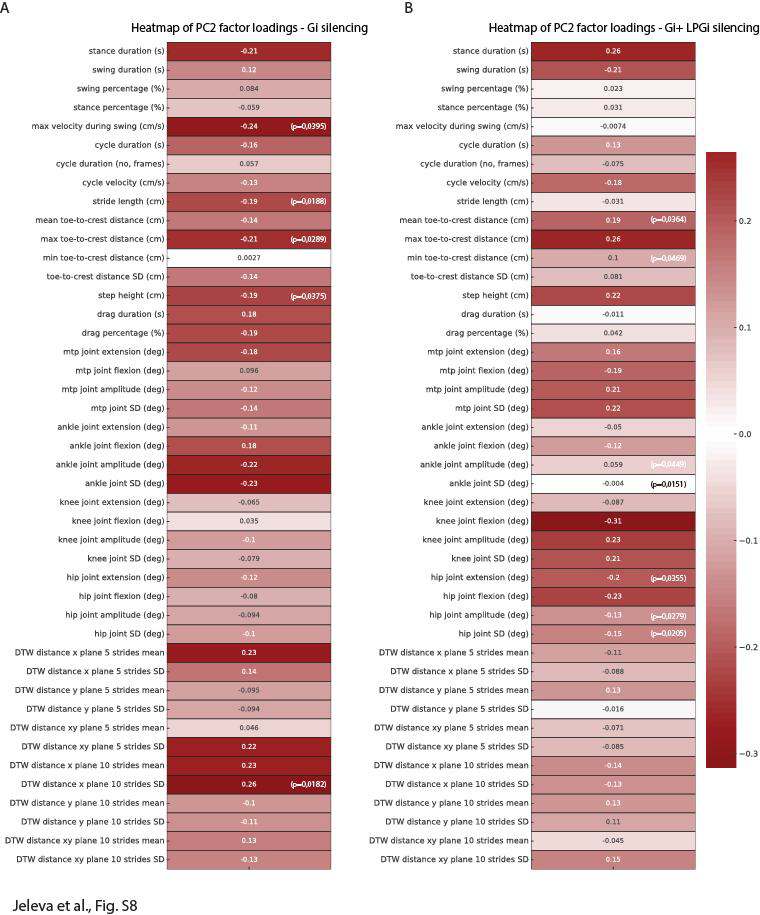

Supplement: Supplementary file 8 [file NRR-21-2011_Suppl8.tif]
